# The Z-cad dual fluorescent sensor detects dynamic changes between the epithelial and mesenchymal cellular states

**DOI:** 10.1186/s12915-016-0269-y

**Published:** 2016-06-17

**Authors:** M. J. Toneff, A. Sreekumar, A. Tinnirello, P. Den Hollander, S. Habib, S. Li, M. J. Ellis, L. Xin, S. A. Mani, J. M. Rosen

**Affiliations:** Department of Molecular and Cellular Biology, Baylor College of Medicine, Houston, TX USA; Department of Translational Molecular Pathology, The University of Texas MD Anderson Cancer Center, Houston, TX USA; Lester and Sue Smith Breast Center, Baylor College of Medicine, Houston, TX USA; Washington University Institute of Clinical and Translational Sciences, St. Louis, MO USA

**Keywords:** EMT, MET, Plasticity, Molecular sensors, Cancer stem cell

## Abstract

**Background:**

The epithelial to mesenchymal transition (EMT) has been implicated in metastasis and therapy resistance of carcinomas and can endow cancer cells with cancer stem cell (CSC) properties. The ability to detect cancer cells that are undergoing or have completed EMT has typically relied on the expression of cell surface antigens that correlate with an EMT/CSC phenotype. Alternatively these cells may be permanently marked through Cre-mediated recombination or through immunostaining of fixed cells. The EMT process is dynamic, and these existing methods cannot reveal such changes within live cells. The development of fluorescent sensors that mirror the dynamic EMT state by following the expression of bona fide EMT regulators in live cells would provide a valuable new tool for characterizing EMT. In addition, these sensors will allow direct observation of cellular plasticity with respect to the epithelial/mesenchymal state to enable more effective studies of EMT in cancer and development.

**Results:**

We generated a lentiviral-based, dual fluorescent reporter system, designated as the Z-cad dual sensor, comprising destabilized green fluorescent protein containing the *ZEB1* 3′ UTR and red fluorescent protein driven by the E-cadherin (*CDH1*) promoter. Using this sensor, we robustly detected EMT and mesenchymal to epithelial transition (MET) in breast cancer cells by flow cytometry and fluorescence microscopy. Importantly, we observed dynamic changes in cellular populations undergoing MET. Additionally, we used the Z-cad sensor to identify and isolate minor subpopulations of cells displaying mesenchymal properties within a population comprising predominately epithelial-like cells. The Z-cad dual sensor identified cells with CSC-like properties more effectively than either the *ZEB1* 3′ UTR or E-cadherin sensor alone.

**Conclusions:**

The Z-cad dual sensor effectively reports the activities of two factors critical in determining the epithelial/mesenchymal state of carcinoma cells. The ability of this stably integrating dual sensor system to detect dynamic fluctuations between these two states through live cell imaging offers a significant improvement over existing methods and helps facilitate the study of EMT/MET plasticity in response to different stimuli and in cancer pathogenesis. Finally, the versatile Z-cad sensor can be adapted to a variety of in vitro or in vivo systems to elucidate whether EMT/MET contributes to normal and disease phenotypes.

**Electronic supplementary material:**

The online version of this article (doi:10.1186/s12915-016-0269-y) contains supplementary material, which is available to authorized users.

## Background

Many cancers, including breast cancer, initially respond to targeted therapy, chemotherapy, or radiotherapy, but the development of resistance to these therapies and subsequent tumor recurrence remain major clinical problems. The majority of breast cancer-related deaths are attributed to metastasis, and metastatic tumors are also associated with therapy resistance [1]. Cells within a particular tumor may display significant phenotypic variability, which can lead to differential responses to therapy. The epithelial to mesenchymal transition (EMT) can endow normal and breast cancer cells with stem cell and cancer stem cell (CSC) properties, respectively [2–4]. Evidence from neoadjuvant breast cancer clinical trials suggests that after chemotherapy or targeted therapy, the surviving tumor cells display a CSC- and EMT-like gene expression profile, suggesting that cancer cells with EMT-like properties can resist these therapies [5]. Moreover, residual cells with an EMT/CSC signature may progress to more aggressive and therapeutically refractory tumors. Further evidence has accumulated in breast and other carcinomas that EMT enables cells to evade conventional treatments [6–10]. These studies highlight the need for strategies to target cells that have undergone an EMT in cancer. Alternatively, strategies that revert mesenchymal cells to a more epithelial state may render them susceptible to standard of care therapies [11].

EMT may also be required in some primary tumors for the metastatic process of invasion and intravasation in which only a small fraction of cells survive, as epithelial cells usually undergo anoikis when they lose contact with the basal lamina [12]. The few surviving cells must then extravasate and colonize secondary organs to establish metastases, where the reverse process, mesenchymal to epithelial transition (MET), may then be required for metastatic cells to proliferate and grow into life-threatening macrometastases [13, 14]. Provocatively, these studies suggest that EMT/MET plasticity itself, and not just a static epithelial or mesenchymal phenotype, plays a major role in pathogenicity. However, the ability of cancer cells to exhibit cellular plasticity with respect to EMT and MET has been difficult to study, as methods that enable direct observations of phenotypic plasticity in live cells are limited. The development of stable sensors of CSC/EMT regulatory factors that mirror dynamic changes in cellular states to isolate, analyze, and observe living cells should allow us to monitor EMT/MET plasticity in real time and improve our understanding of these processes.

To better identify mesenchymal-like cells or cells undergoing EMT among a heterogeneous population, cells that may be resistant to therapy and mediate metastasis, we developed lentivirus-based fluorescent sensors that stably integrate into a cell’s genome and report changes in bona fide regulators of EMT. In this study we generated a dual fluorescent sensor system, designated as the Z-cad dual sensor system, which comprises two individual sensors: a constitutively expressed, destabilized green fluorescent protein (GFP) reporter regulated by the *ZEB1* 3′ untranslated region (UTR) and a red fluorescent protein (RFP) reporter driven by the E-cadherin (*CDH1*) promoter. ZEB1 is a potent inducer of EMT and mediates its effects in part through direct suppression of E-cadherin transcription [15]. ZEB1 forms a double-negative feedback loop with the miR-200 family of microRNAs, which induce MET by binding to the *ZEB1* 3′ UTR, thus inhibiting *ZEB1* translation [16–19]. E-cadherin is a common epithelial effector molecule that mediates epithelial cell interactions, and inhibition of its expression is associated with EMT [20]. Here we validated the function of these sensors by identifying MET from mesenchymal-like breast cancer and conversely EMT from epithelial-like cells. In addition we used these sensors to successfully isolate cells with EMT and CSC properties from a heterogeneous population. Importantly, we were able to identify changes over time in a transitioning population using fluorescent microscopy, demonstrating the ability to observe dynamic changes from the mesenchymal to the epithelial state. Finally, we show that a subset of cells that have permanently undergone EMT, as identified by their Z-cad sensor fluorescence pattern and morphology, can be forced to undergo MET through epigenetic reprogramming using a DNA methyltransferase inhibitor.

## Results

### Construction and validation of fluorescent EMT sensors

**Fig. 1:**
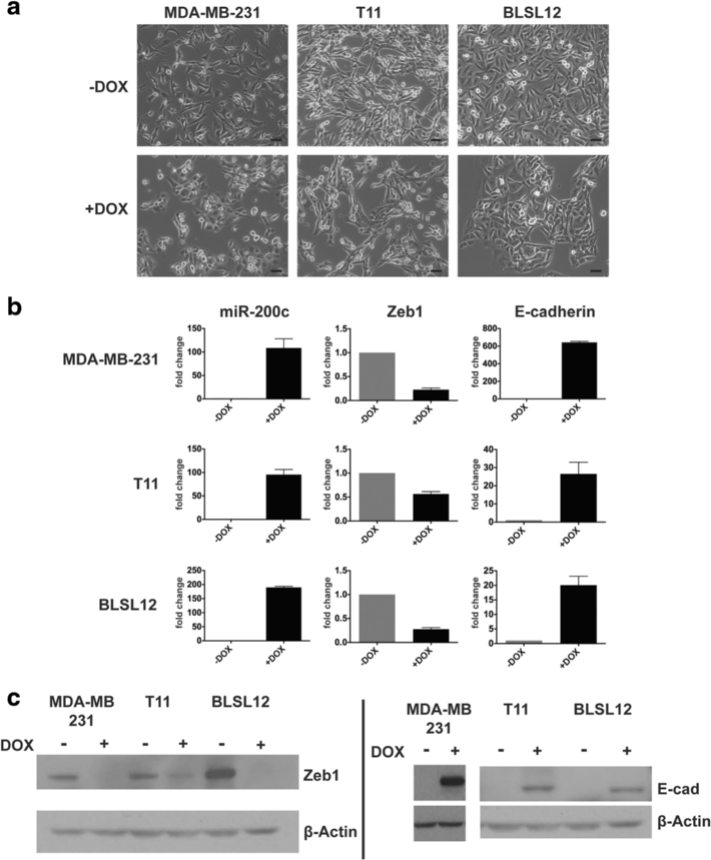
miR-200c/141 expression elicits MET in claudin-low breast cancer. **a** MDA-MB-231, T11, and BLSL12 cells treated with 2 μg/mL doxycycline (+DOX) for 4 days undergo morphological MET (*n* = 3 biological replicates). Scale bar = 20 μm. **b** qRT-PCR analysis of indicated genes after 4 days of DOX treatment, *n* = 3. **c** Western blot analysis for indicated proteins after 4 days of DOX treatment

To establish inducible models that alter the EMT state of carcinoma cells, we selected three mesenchymal-like, claudin-low breast cancer models: the human MDA-MB-231 cell line, the mouse T11 cell line [21], and the human BLSL12 breast cancer cell line derived from the WHIM12 patient-derived xenograft (PDX) [22]. To induce MET in these cells, we transduced each cell line with the pINDUCER lentivirus [23] containing the doxycycline-inducible human miR-200c/141 cluster (miR-200c), followed by selection for provirus-positive cells. We confirmed that the mesenchymal-like claudin-low cells switch to an epithelial-like (MET) morphology upon miR-200c induction as compared to non-induced cells (Fig. 1a). Induction of miR-200c (+DOX) was confirmed by qRT-PCR in each cell line (Fig. 1b). MET was further confirmed by reduced ZEB1 expression and increased E-cadherin expression in each cell line by qRT-PCR and western blot analysis (Fig. 1b–c).

**Fig. 2:**
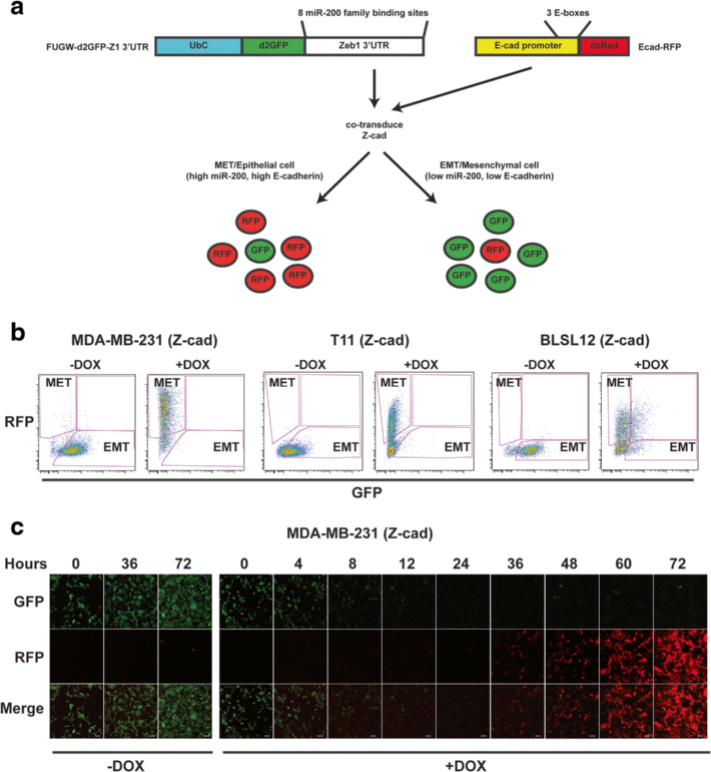
The Z-cad dual sensor detects MET in claudin-low human and mouse breast cancers. **a** (*Left*) The d2GFP-Z1 3′ UTR sensor constitutively expresses destabilized GFP (d2GFP) under control of constitutively active ubiquitin C promoter (UbC). The human *ZEB1* 3′ UTR containing eight miR-200 binding sites was cloned downstream of d2GFP. (*Right*) The Ecad-RFP sensor contains about 1370 bp of the human E-cadherin promoter regulating dsRED (RFP) expression. Three E-boxes proximal to transcription start site are indicated. Co-transduction of both sensors yields cells containing the Z-cad dual sensor. **b** Flow cytometric analysis of human MDA-MB-231 breast cancer cells, mouse T11 breast cancer cells, and human BLSL12 PDX-derived cells containing the Z-cad sensor. miR-200c/141 was induced with 2 μg/mL doxycycline for 4 days and flow cytometry was performed. Epithelial (MET)- and mesenchymal (EMT)-enriched populations are as indicated (*n* = 3 biological replicates per group). **c** Fluorescent confocal microscopy of MDA-MB-231 cells imaged at indicated time points during induction (+DOX) of miR-200c. –DOX is non-induced control. Scale bar = 50 μm

To generate stable fluorescent sensors that can identify carcinoma cells with EMT or MET properties, we used the lentiviral expression vector FUGW, which expresses eGFP from the constitutively active ubiquitin C (UbC) promoter [24]. The human *ZEB1* 3′ UTR, a direct target of miR-200 family members containing eight miR-200 target sequences [25], or a 3′ UTR containing five miR-200 target sequences was inserted downstream of GFP (Fig. 2a and Additional file 1: Figure S1A). It is important to note that the *ZEB1* 3′ UTR sensor *does not* report transcriptional activity of the *ZEB1* promoter, but instead reports post-transcriptional regulation of *ZEB1* via its 3′ UTR. The eGFP fluorescent protein has a stability of >24 hours [26], which prevents rapid detection of decreasing GFP protein expression. Because we were interested in detecting rapid changes in GFP in response to changes in miR-200 family member activity (e.g., GFP^hi^ to GFP^low/neg^), we replaced eGFP with a destabilized GFP (d2GFP), which has a half-life of about 2 hours [27]. We designated the sensor using the human *ZEB1* 3′ UTR as d2GFP-Z1 3′ UTR and the 3′ UTR containing five miR-200 target sequences as d2GFP-200. Use of these sensors in mesenchymal-like cells and cells undergoing an EMT, which express low levels of miR-200 family members, should result in high GFP expression. Conversely, their use in epithelial-like cells or cells undergoing an MET, which have increased miR-200 expression, should result in low GFP expression by attenuation of GFP translation (Fig. 2a). Therefore, these GFP-based sensors should identify EMT (or mesenchymal cells) versus MET (or epithelial cells) states as a function of changing miR-200 levels.

Although our GFP-based sensors report a single aspect of EMT regulation (namely miR-200 expression and other regulators of *ZEB1* via its 3′ UTR), many other factors can determine an epithelial versus mesenchymal-like state. The use of multiple detectors of EMT should provide greater resolution in identifying changes in cellular state. The human E-cadherin (*CDH1*) promoter contains three proximal E-boxes that serve as binding sites for E-cadherin transcriptional repressors including ZEB1, ZEB2, Snail, and Slug [28]. E-cadherin is not expressed in the mesenchymal state, and its loss promotes EMT and CSC properties [29]. Therefore, to detect the presence of an addition regulator of an epithelial versus mesenchymal state, we replaced the cytomegalovirus (CMV) promoter in a pHAGE lentiviral vector that expresses dsRed [30] with the E-cadherin promoter. We have designated this as the Ecad-RFP sensor (Fig. 2a). Simultaneous introduction of the GFP- and RFP-based sensors, therefore, should allow robust identification of both epithelial- and mesenchymal-like cells. Furthermore, this dual sensor approach should provide an additional level of specificity by helping to distinguish general effects on transcription and microRNA biogenesis.

To test the function of these sensors, each claudin-low cell line containing inducible miR-200c was co-transduced with the d2GFP-Z1 3′ UTR and the Ecad-RFP sensors, which, when used in combination, we have designated as the Z-cad dual sensor. If the sensors function properly in cells containing the Z-cad dual sensor, miR-200c should directly suppress GFP (and endogenous *ZEB1*) expression via the *ZEB1* 3′ UTR. Reduction of endogenous *ZEB1*, in turn, should elicit de-repression of RFP regulated by the E-cadherin promoter (Fig. 2a). We induced miR-200c expression and performed flow cytometry analysis for GFP and RFP. The non-induced cells expressed GFP and little to no RFP, the expected fluorescent characteristics of EMT cells harboring the Z-cad dual sensor, in MDA-MB-231, T11, and BLSL12 cells (Fig. 2b, –DOX). However, in each cell line tested, miR-200c induction inhibited GFP expression and induced RFP expression from the Z-cad dual sensor, the expected fluorescent characteristics of MET cells, validating the ability of these sensors to successfully report MET (Fig. 2b, +DOX). We saw a similar effect in each cell line with combinatorial use of d2GFP-200/Ecad-RFP (Additional file 1: Figure S1C–D). Control GFP expression was not reduced upon miR-200c induction as shown in Additional file 1: Figure S1C–D.

To demonstrate the ability of the Z-cad dual sensor to detect dynamic changes in response to stimuli affecting the mesenchymal state, we treated MDA-MB-231 cells containing the Z-cad dual sensor with DOX to induce miR-200c and imaged the cells over the course of 72 hours. We first observed loss of GFP expression between 4 and 6 hours (Additional file 2: Figure S2B), and by 12 hours, almost all GFP expression was non-detectable, whereas no changes in GFP fluorescence levels were observed in the control (–DOX) cells (Fig. 2c and Additional file 2: Figure S2A). RFP was first observed to increase beginning at 32 hours (Additional file 2: Figure S2B) and continued to increase in intensity throughout the 72 hours of DOX induction of miR-200c (Fig. 2c and Additional file 2: Figure S2B). These results demonstrated that in response to miR-200c expression, GFP expression was rapidly lost and there was a delayed but significant increase in RFP expression, as would be expected because E-cadherin is an indirect downstream target of miR-200c. The ability of the Z-cad sensor to detect these dynamic changes is a significant improvement over analysis of cells by immunostaining or via flow cytometry at a single time point. Importantly, the changes in GFP and RFP reflected the changes in ZEB1 and E-cadherin mRNA and protein levels (Fig. 1), respectively, demonstrating that expression of these fluorescent sensors reflected the expression of their endogenous counterparts. We additionally confirmed the changes in Z-cad fluorescence by microscopy in the mouse T11 and human PDX-derived BLSL12 cells 4 days after DOX treatment (Additional file 1: Figure S1B and E). Therefore, using the Z-cad dual sensor system, we were able to detect changes in the mesenchymal state upon MET induction in both human and mouse breast cancer cells.

### The Z-cad dual sensor identifies EMT induced by TGFβ1 in HMLER cells

**Fig. 3:**
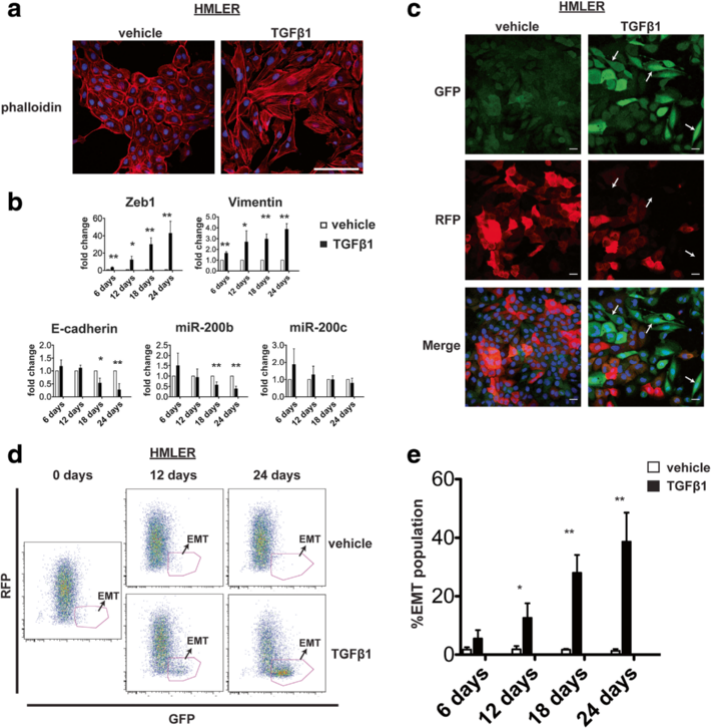
The Z-cad sensor identifies EMT induced by TGFβ1 in HMLER cells. **a** F-actin stress fibers increase during TGFβ1 compared to vehicle treatment (26 days) indicating EMT. Scale bar = 100 μm. **b** RNA analysis by qRT-PCR at indicated time points during vehicle or TGFβ1 treatment (*n* = 3 biological replicates per time point). Vehicle-treated values were set to 1.0 for each gene and analyzed using an unpaired Student’s *t* test. **c** Fluorescent confocal microscopy indicating a gain of GFP and loss of RFP at 14 days of TGFβ1 versus vehicle treatment. Mesenchymal-appearing bright GFP cells are indicated by *arrows*. Scale bar = 20 μm. **d** Flow cytometric analysis of HMLER cells containing Z-cad sensor showing an increase in cells displaying GFP^hi^RFP^low/neg^ EMT fluorescence signature during TGFβ1 treatment at the times indicated. **e** Quantitation of cells displaying the EMT signature in HMLER cells containing Z-cad dual sensor from **d**. Bar graphs depict the percentage of cells displaying the EMT fluorescence signature in TGFβ1- versus vehicle-treated groups (*n* = 3 biological replicates per time point). Data were analyzed using an unpaired Student’s *t* test. * *p* value < 0.05, ** *p* value < 0.01

Having validated the use of the Z-cad dual sensor system to detect EMT/MET in claudin-low breast cancer cells expressing inducible miR-200c, we next asked if we could identify cells with EMT/MET properties using the Z-cad dual sensor in response to TGFβ1. For this, we used the epithelial-like, experimentally transformed human mammary epithelial cell line, HMLER [31]. HMLER cells containing the Z-cad sensor were treated with TGFβ1 (5 ng/mL) to induce EMT or vehicle over the course of several days, as it took this time for the cells to undergo an epithelial to mesenchymal switch. At the end of the treatment, cells in the TGFβ1-treated group had a high incidence of F-actin stress fibers, compared with few stress fibers in the vehicle-treated cells, consistent with cells exhibiting an EMT phenotype (Fig. 3a). We harvested TGFβ1- versus vehicle-treated cells at several time points throughout a 24-day treatment period and performed qRT-PCR for EMT-associated genes. Analysis of *ZEB1*, vimentin, E-cadherin*,* miR-200b, and miR-200c expression levels demonstrated an early induction of the mesenchymal-associated genes *ZEB1* and vimentin (by 6 days) followed by a reduction of the epithelial-associated genes E-cadherin and miR-200b (Fig. 3b). We did not observe a significant reduction in miR-200c RNA during the time course of the experiment (Fig. 3b).

Next, we asked whether the observed changes in gene expression were reflected by Z-cad dual sensor expression. We noted that HMLER cells not treated with TGFβ1 displayed a wide range of RFP by flow cytometry analysis, although the majority did express RFP (90 ± 3.0 % RFP-positive cells; *n* = 8), suggesting that there is heterogeneity in HMLER cells with respect to E-cadherin expression (see Fig. 5). At day 14 of treatment, TGFβ1- or vehicle-treated cells were assessed for differences in fluorescence by microscopy. The majority of vehicle-treated cells showed weak GFP expression and a wide range of RFP levels (from weak to strong), whereas the TGFβ1-treated cells displayed brighter GFP expression with fewer RFP-expressing cells (Fig. 3c). In addition, the high GFP-expressing cells in the TGFβ1-treated group frequently appeared mesenchymal in morphology (Fig. 3c, arrows). Flow cytometry analysis of the cells throughout the 24-day treatment period showed a gradual increase in the proportion GFP^hi^/RFP^low/neg^ (EMT signature) cells in the TGFβ1- compared to vehicle-treated cells (Fig. 3d–e). The observed changes in gene expression (Fig. 3b) reflected the increasing EMT population observed by flow cytometry, demonstrating that the Z-cad dual sensor was able to identify cells that have undergone EMT in response to TGFβ1.

### The Z-cad dual sensor identifies differential plasticity in EMT subpopulations of HMLER cells upon TGFβ1 exposure

**Fig. 4:**
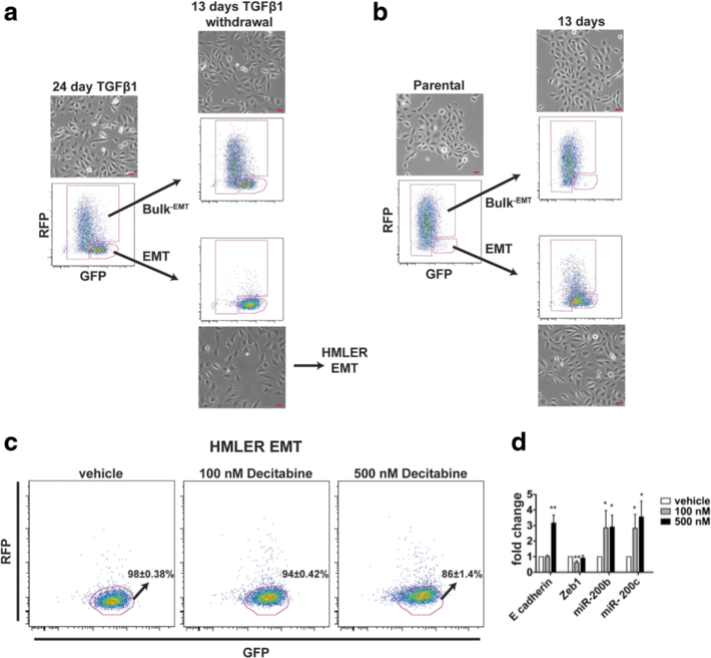
The Z-cad sensor identifies differential plasticity in HMLER EMT subpopulations upon TGFβ1 exposure. **a** HMLER cells cultured for 24 days with TGFβ1 were sorted into EMT and Bulk^–EMT^ groups and allowed to grow in the absence of TGFβ1 for 13 days. **b** Parental HMLER cells were grown sorted into EMT and Bulk^–EMT^ groups and allowed to grow for 13 days. **c** HMLER EMT cells were grown in the presence of the indicated concentrations of decitabine (or vehicle) for 5 days. Medium was changed daily with fresh decitabine. Flow cytometry was performed and the EMT fluorescence signature is indicated (*n* = 3 biological replicates for vehicle and 500 nM; *n* = 2 biological replicates for 100 nM). **d** qPCR analysis for the indicated genes at different decitabine concentrations is shown. Vehicle treated values were set to 1.0 for each gene. Each concentration was compared to vehicle and analyzed using an unpaired Student’s *t* test (*n* = 3 biological replicates per group). * *p* value < 0.05, ** *p* value < 0.01. All scale bars = 20 μm

To assess the plasticity of cells following TGFβ1 treatment, we used fluorescent activated cell sorting (FACS) to separate TGFβ1-treated cells into two populations, the EMT signature cells and the bulk population depleted of the EMT signature cells (Bulk^-EMT^) at 12 (data not shown) or 24 days of treatment, and then, each population was cultured for 13 days in the absence of TGFβ1. Intriguingly, following 13 days of TGFβ1 removal, the EMT signature cells were mesenchymal in appearance and maintained their EMT fluorescence signature (GFP^hi^/RFP^low/neg^), whereas the Bulk^-EMT^ cells appeared only partially mesenchymal and displayed a heterogeneous fluorescence signature (Fig. 4a). These data suggest that only a subset of the parental HMLER cells undergo complete EMT during TGFβ1 treatment, whereas the majority of cells, even at late time points, are still transitioning and display a partial mesenchymal phenotype. The EMT signature sorted cells treated with TGFβ1 displayed no capacity to revert to their parental phenotype, both morphologically and by Z-cad expression, upon subsequent passaging after 13 days (data not shown). However, FACS-isolated parental HMLER cells that displayed the EMT signature and were never exposed to TGFβ1 eventually re-expressed RFP, lost GFP expression, and lacked the full mesenchymal morphology (Fig. 4b). This suggested that parental HMLER cells that display the EMT signature retain plasticity, as they have the capacity to undergo MET. Together, these data indicate that TGFβ1 treatment reprograms a subset of HMLER cells and inhibits their capacity for EMT/MET plasticity.

HMLE cells, from which the HMLER cells are derived, undergo spontaneous EMT with concomitant methylation of miR-200c, re-expression of which induces MET [32]. Therefore, we hypothesized that prolonged exposure of cancer cells to TGFβ1 potentially induces DNA methylation and fixes cells in the mesenchymal state. To test this hypothesis, we treated fully transitioned HMLER EMT cells with the DNA methyltransferase inhibitor decitabine for 5 days. Decitabine treatment reduced the number of cells displaying the Z-cad EMT signature compared to vehicle-treated cells (Fig. 4c). Quantitation of RFP expression showed a significant increase in RFP^+^ cells from 1.09 % in vehicle-treated cells to 2.84 % (*p* = 0.006) and 5.33 % (*p* = 0.003) for 100 nM and 500 nM decitabine-treated cells, respectively (Additional file 3: Figure S3). Moreover, we observed a significant increase in GFP^low^ expressing cells in vehicle-treated cells from 4.63 % to 8.07 % (*p* = 0.007) and 15.7 % (*p* = 0.0000002) for 100 nM and 500 nM decitabine treated cells, respectively (Additional file 3: Figure S3). Decitabine-treated cells acquired the expression of E-cadherin, miR-200b, and miR-200c, and reduced *ZEB1* expression (Fig. 4d). Thus, the changes in the Z-cad sensor reflect the changes in the endogenous expression of these genes. These data further suggest that the permanent EMT induced by TGFβ1 treatment may be due, at least in part, to epigenetic silencing of epithelial genes by DNA methylation, and this can be reversed, in at least a subset of cells, by DNA methyltransferase inhibition.

### Combinatorial use of EMT sensors detects rare mesenchymal-like cells among a heterogeneous population largely comprising epithelial-like cells

**Fig. 5:**
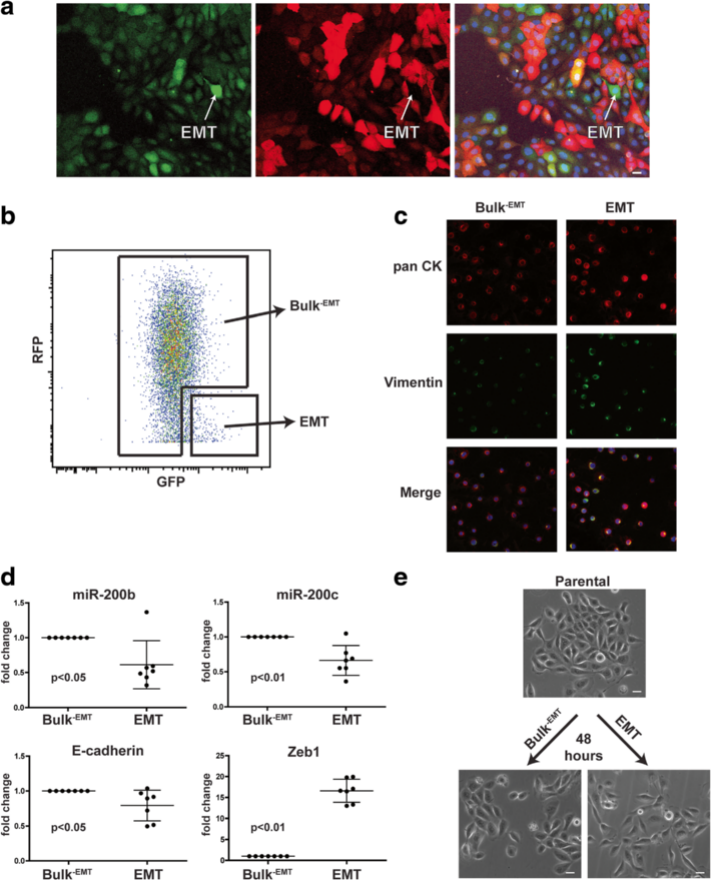
The Z-cad sensor identifies and enables isolation of mesenchymal cells present among a heterogeneous population. **a** Fluorescent confocal microscopy was performed on parental HMLER cells containing the Z-cad dual sensor. Scale bar = 20 μm. **b** FACS was performed on parental HMLER cells to isolate mesenchymal-like GFP^hi^RFP^low/neg^ cells, which comprise the EMT signature. All other cells are designated as Bulk^–EMT^. **c** Cells from each group in **b** were collected, cytospun, and stained for pan-CK and vimentin. **d** RNA from each group collected in **b** was isolated and qRT-PCR performed for the indicated genes. Bulk^–EMT^ cell values were set to 1.0 for each gene. Data were analyzed using a paired Student’s *t* test (*n* = 7 biological replicates). **e** Cells isolated from each group in **b** were plated and grown for 48 hours. Scale bar = 20 μm

It is apparent that considerable heterogeneity exists in most established epithelial cell lines. Parental HMLER cells cluster into the basal-like breast cancer subtype and contain a small population of CD24^low/neg^/CD44^hi^ CSCs that also display EMT properties [2, 33]. In our studies using HMLER cells, TGFβ1 treatment slowly induced EMT, and the transition only appeared to be complete in a subset of cells. Notably, the Z-cad dual sensor allowed the resolution of cells displaying a partial versus complete EMT during TGFβ1 treatment, suggesting that there is significant heterogeneity within the HMLER population during TGFβ1-induced EMT. Therefore, we next asked whether parental HMLER cells display epithelial/mesenchymal heterogeneity similar to that observed during TGFβ1 induction of EMT, and if the Z-cad dual sensor can resolve distinct cellular populations prior to experimental induction of EMT. Using fluorescent microscopy, we observed differences in the intensity of fluorescence among individual cells, and we identified a small subpopulation of GFP^+^RFP^neg^ cells that appeared mesenchymal in morphology (Fig. 5a). Flow cytometry analysis (Fig. 5b) of parental HMLER cells also showed a group of GFP^hi^RFP^low/neg^ cells, similar to the EMT signature cells that expanded during TGFβ1 treatment (Fig. 3). We asked whether cells falling within this EMT signature group in parental HMLER cells display characteristics of mesenchymal cells even without experimental EMT induction. Using FACS we separated cells displaying the EMT signature (GFP^hi^RFP^low/neg^) from the Bulk^-EMT^ cells. We cytospun the cells immediately post sorting and performed co-immunofluorescence for pan-cytokeratin (CK), an epithelial marker, and vimentin, a mesenchymal marker. Although all cells expressed basal levels of both CK and vimentin, the immunofluorescent staining of vimentin was more intense in the EMT signature associated cells relative to the Bulk^–EMT^ population (Fig. 5c). Moreover, RNA analysis showed reduced expression of E-cadherin*,* miR-200b, and miR-200c and increased expression of *ZEB1* in the EMT signature population relative to the Bulk^–EMT^ population (Fig. 5d). We also used the Z-cad dual sensor system in MCF10A cells, which have been shown to also be heterogeneous and contain a more mesenchymal subpopulation [34], and observed a similar RNA expression pattern when sorted into EMT and Bulk^–EMT^ populations to that observed in the HMLER cells (Additional file 4: Figure S4). Finally, sorted HMLER cells were cultured and their morphology was assessed 48 hours post sorting. Bulk^–EMT^ cells appeared cobblestone-like and formed tightly organized colonies, similar to the unsorted, parental HMLER cells (Fig. 5e). Conversely, the EMT signature cells displayed a loosely packed organization and were more mesenchymal in morphology than the parental cells (Fig. 5e), but similar to the sorted parental EMT signature cell morphology observed 13 days after sorting (Fig. 4b). Taken together, these data indicate that the Z-cad dual sensor can be used to identify and isolate cellular subpopulations that display mesenchymal-like morphology and gene expression from a heterogeneous population of cells.

During TGFβ1 treatment of HMLER cells (Fig. 3), we observed a gradual induction of a complete EMT via formation of a GFP^hi^RFP^low/neg^ population, and these cells were more mesenchymal in morphology than the Bulk^-EMT^ population (Fig. 4a). While the entire TGFβ1-treated cell population gained expression of mesenchymal genes early on compared to the vehicle-treated cells, a decrease in epithelial gene expression was delayed (Fig. 3b). However, the separation of cells with the EMT signature from the Bulk^–EMT^ population resolved differences in RNA and protein expression with respect to EMT regulating factors during TGFβ1 treatment (Additional file 5: Figure S5). This validated the ability of the Z-cad dual sensor to identify changes in gene expression among a heterogeneous population even when these changes were not apparent among the entire TGFβ1-treated population as compared to the vehicle-treated population until later time points. Taken together, these data demonstrate that the Z-cad dual sensor can be used to isolate and identify different cellular populations within a heterogeneous population displaying more mesenchymal gene expression patterns and properties.

### Z-cad dual fluorescence better identifies CSC-like cells compared with either sensor alone

**Fig. 6:**
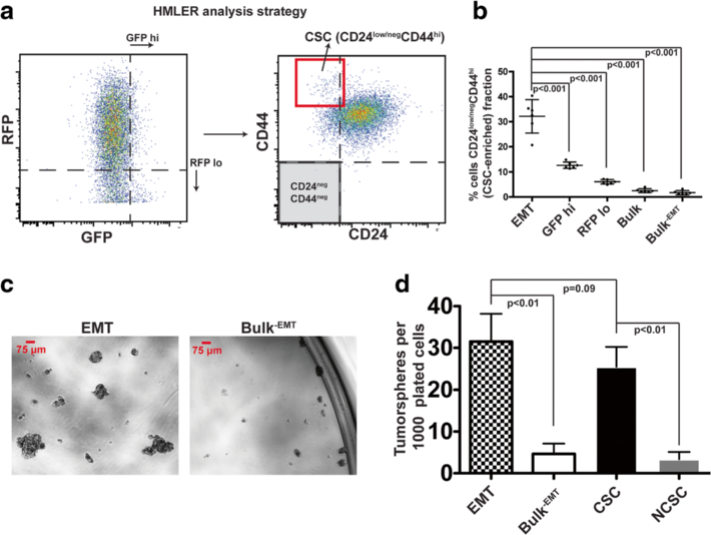
The Z-cad dual sensor enriches for CSCs better than either sensor alone. **a** Analysis strategy for identifying CD24/CD44 expression of HMLER cells first gated based on distinct Z-cad expression patterns. The CD24/CD44 plot shown here indicates the entire cellular population (Bulk) without prior Z-cad gating. CD24 and CD44 negative box is shown in *gray* and was identified using singly stained samples. CSC-enriched population (CD24^low/neg^/CD44^hi^) is shown in the *red box*. **b** Percentage of cells falling into CSC-enriched population (CD24^lo/neg^CD44^hi^) first gated on indicated Z-cad sensor fluorescence patterns. Bulk represents all cells. Data were analyzed using the paired Student’s *t* test (*n* = 6 biological replicates). **c** HMLER cells were sorted into EMT and Bulk^–EMT^ groups (shown) or CSC and NCSC (not shown) and were plated and grown under tumorsphere conditions (1000 cells per well in a 96-well plate, 5 technical replicates per biological replicate, 3 biological replicates). **d** After 12 days, spheres >75 μm were quantified. Data were analyzed using the paired Student’s *t* test

Previous studies have demonstrated that HMLE cells contain a subpopulation of CSCs as demonstrated by their CD24^low/neg^CD44^hi^ expression and mammosphere-forming capacity [2, 32]. Because EMT has been linked to CSC properties, we asked how well the HMLER cells displaying an EMT signature, which were isolated using the Z-cad dual sensor system from the parental HMLER cells, correlated with HMLER cells displaying a CSC signature (CD24^low/neg^/CD44^hi^). Moreover, we wanted to test whether the Z-cad sensor system could better identify CSCs than either sensor alone. We used flow cytometry to distinguish cells displaying the GFP^hi^RFP^low/neg^ EMT signature to those displaying GFP^hi^, RFP^low/neg^, Bulk^-EMT^, and the total cellular population (Bulk), and then quantified the percentage of these populations that fell within the CSC-enriched CD24^low^/CD44^hi^ population (Fig. 6a–b; Additional file 6: Figure S6A–B). The GFP^hi^ only and the RFP^low^ only populations contained more cells in the CD24^low/neg^/CD44^hi^, CSC-enriched group than the Bulk population (13 % versus 2.5 %, *p* = 0.0000037) and (6.1 % versus 2.5 %, *p* = 0.00052), respectively, demonstrating that either sensor alone can enrich for CSCs. Importantly, the EMT signature population was the most highly enriched in the CD24^low^/CD44^hi^ CSC signature group compared with the Bulk population (32 % versus 2.5 %, *p* = 0.00018) (Fig. 6b; Additional file 6: Figure S6B), and compared with any of the other populations, including GFP^hi^ (*p* = 0.00074), RFP^low^ (*p* = 0.00017), and Bulk^–EMT^ (*p* = 0.00017) (Fig. 6b and Additional file 6: Figure S6B). These data suggest that the Z-cad sensor better enriches for CSCs than either sensor alone.

To test the stem cell function of the EMT signature cells isolated using the Z-cad sensor, we performed tumorsphere assays. Importantly, the EMT signature cells formed tumorspheres (>75 μm) at a frequency of about 3 %, much higher than the approximately 0.5 % tumorsphere-forming capacity of the Bulk^-EMT^ population (Fig. 6c–d). In addition, cells falling into the CD24^low/neg^/CD44^hi^ CSC-enriched population formed tumorspheres at a higher rate than the non-CSC (NCSC) population (Fig. 6d). Confirming the ability of the Z-cad sensor to identify cells with CSC properties, cells with the EMT signature formed spheres at a similar efficiency as that of the CSC-enriched population (Fig. 6d). These data, therefore, demonstrated that the Z-cad dual sensor facilitated the isolation of cells with EMT properties from a mixed population, and furthermore, it was able to enrich for cells with CSC properties to a greater extent than either sensor alone. Therefore, the Z-cad dual sensor should provide a stable and facile method, in the absence of immunostaining, for identifying and isolating EMT/CSC-like cells by flow cytometry in this model as well as for their detection via microscopy.

## Discussion

In this study we have described the development and validation of novel fluorescent reporter-based sensors for the identification of an EMT/MET state. We also demonstrated that the Z-cad dual sensor successfully reports the regulation of two critical regulators of EMT. One component of our Z-cad dual sensor system, the *ZEB1* 3′ UTR sensor (and miR-200 sensor), reports the activity of the miR-200 family of microRNAs. ZEB1 and the miR-200 family represent a critical axis that determines epithelial/mesenchymal identity and may be responsible for EMT/MET plasticity [35, 36]. The ZEB1/miR-200 axis likely has additional, non-cell autonomous roles in cancer pathogenesis, as it has recently been shown to affect immune recognition of cancer cells whereby ZEB1 suppression of the miR-200 family leads to upregulation of PD-L1, a direct miR-200 family target. This leads to subsequent evasion of immune cells by the tumor cells [37]. The study by Chen et al. [37] suggests an ever-expanding role of EMT in regulating different aspects of cancer and highlights the need to more fully understand the critical ZEB1/miR-200 axis. The second component of our Z-cad dual sensor system is a promoter-based sensor that reports transcriptional regulation of E-cadherin, the expression of which is lost during EMT. This loss is mediated through direct transcriptional suppression by ZEB1 and other inducers of EMT, including ZEB2, Snail, and Slug, that target E-boxes present in the E-cadherin promoter [20]. One potential caveat is that the E-cadherin reporter component of this sensor may not detect EMT in cases where E-cadherin is post-transcriptionally regulated or its localization at the cell membrane is lost, which can also induce EMT properties [38]. However, the GFP component of the Z-cad sensor in these cases would still report the ZEB1/miR-200 axis. Additionally, it is not inconceivable that the Z-cad sensor will have limited applicability in detecting EMT regulated independently of miR-200, ZEB1, or E-cadherin. Indeed, miR-200 and ZEB1 expression are highly intertwined, and ZEB1 does regulate E-cadherin expression. However, a number of other transcription factors including ZEB2, Slug, and Snail can also suppress E-cadherin expression; therefore, E-cadherin expression is not solely dependent upon ZEB1. Nevertheless, the prevalence of the ZEB1/miR-200 axis and E-cadherin in regulating EMT suggests that the Z-cad sensor provides a widely applicable tool for studying the role of EMT and MET in cancer.

Our data demonstrate that the Z-cad sensor system can be employed to determine whether EMT or MET has occurred within heterogeneous populations of both human and mouse cells and should facilitate the identification and isolation of individual cells displaying EMT/MET within a heterogeneous population. While not investigated directly in this study, the Z-cad sensor should allow the detection of cells displaying a partial EMT (e.g., GFP^hi^RFP^hi^), a state that is proposed to enhance the invasive and CSC properties of cancer [39]. The potential for the Z-cad sensor to detect different degrees of EMT should aid in the study of a partial EMT phenotype and its role in cancer pathogenesis. The lentiviral backbone of these sensors facilitates the introduction of stably integrated reporters into diverse tumor models including transplantable models for in vivo studies. This represents a benefit over conventional transgenic lineage tracing models, and has potential utility in developmental studies, including embryonic mammary gland development, a stage when EMT has been hypothesized to occur [40].

A common method for detecting EMT/MET is by using flow cytometry to correlate the EMT phenotype with expression of cell surface antigens associated with CSCs, including high CD44 expression in conjunction with low CD24 or EPCAM expression. Although CD44 may regulate an EMT/CSC state, its specific function in this process and whether its expression is a cause of or a consequence of EMT/CSCs remain controversial [41]. Most likely certain alternatively spliced isoforms of CD44 regulate the EMT/CSC properties of a cell, but often the CD44 antibodies most commonly used in flow cytometry studies to detect EMT/CSCs are not isoform specific [42]. Importantly, the Z-cad sensor used in this study reports expression of two critical regulators of EMT with well-defined roles in the process. The endogenous nature of the Z-cad sensor, once introduced into cells, obviates the need for immunostaining, leading to reductions in cost and time when performing these analyses. Here we confirmed that the Z-cad dual sensor enriched for cells with a CSC-enriched surface antigen profile better than either component of the Z-cad sensor alone. However, while the correlation between EMT and CSC surface antigens has proven useful and relatively accurate here and in other studies, CSCs do not necessarily exhibit EMT properties, and EMT may not always induce CSC properties [43, 44]. Thus, the Z-cad sensor should provide an accurate indicator of EMT as regulated by the ZEB1/miR-200 axis and E-cadherin transcription, and its use will complement surface antigen analysis in cases where CSC frequency and the EMT phenotype do not correlate.

Lineage tracing of cells that have undergone an EMT or MET has relied upon Cre-mediated recombination in cells that express EMT-associated genes [9, 45, 46]. A recent study by Zhao et al. demonstrated the ability to observe EMT in vivo by live imaging using an *FSP1-Cre* transgenic mouse breast cancer model in which the authors were able to observe EMT within individual cells [46]. However, Cre-based models lack the ability to observe true plasticity between the EMT/MET states during live imaging, as Cre-recombination is permanent and only indicates whether a single transition occurred. A recent study used an inducible Twist1 model to show that a transient EMT must occur for CSC formation, followed by a subsequent MET that promoted CSC characteristics [47], emphasizing the relevance of monitoring transient changes in EMT/MET states. Importantly, we have demonstrated that the Z-cad sensor allowed for the analysis of dynamic fluctuations between an EMT and MET state in cell culture models, and this technique should be adaptable to in vivo imaging models, highlighting a significant improvement in the ability to observe EMT/MET plasticity in real time.

While both established breast cancer cell lines and primary tumors exhibit cellular heterogeneity with respect to EMT/CSC properties, and it has been shown that EMT/CSC characteristics are enriched following treatment with breast cancer therapies [5, 48], it is not known whether these therapies induce an EMT or simply select for cells already displaying EMT properties. Conversion from a non-CSC to CSC-like identity has been demonstrated and is accompanied by changes in EMT markers [33, 49]. This conversion may serve as a mechanism for non-CSCs that are normally susceptible to therapy to become more resistant, mesenchymal-like cells. EMT is accompanied by a significant reduction in proliferation [13, 50], suggesting that the transition itself may endow a cell with resistance to therapies that target proliferating cells. However, this is most likely not the only mechanism of resistance, as mesenchymal-like claudin-low breast cancers, which are proliferative, also display characteristics associated with therapy resistance, suggesting that alternative resistance mechanisms exist [5, 51]. Perhaps EMT/CSCs are better able to repair their DNA in response to damage, as has recently been shown for CSCs in a p53 null breast cancer model [52]. Ultimately, the Z-cad sensor may be useful to better address the question of whether plasticity itself provides a resistance mechanism or, alternatively, if preselection of resistant EMT-like cells results in therapy resistance.

Experimental evidence has pointed to EMT/MET plasticity as a requirement for different steps in the metastatic cascade [13, 14], but these studies typically have relied upon overexpression of EMT regulatory factors and therefore, may not reflect what happens in cells that activate or suppress endogenous EMT regulatory factors. Two recent studies suggested that EMT does not increase metastatic potential, highlighting the controversy surrounding the role of EMT in metastasis. However, both studies also reported that EMT still endowed cells with therapy resistance in primary and metastatic tumors [9, 10]. Although the data presented indirectly suggested that EMT/MET plasticity occurs in the resistant metastatic breast tumors, whether the resistant cells actually exhibited plasticity needs to be confirmed. Definition of the precise role of EMT in metastasis, therefore, requires further study. Nevertheless, EMT appears to be important in the pathogenesis of cancer. Here, we provide evidence that the Z-cad sensor can be used to isolate cells in a heterogeneous population without experimentally inducing/inhibiting an EMT, demonstrating its ability to report endogenous expression of EMT regulating factors. Moreover, the Z-cad dual sensor offers a promising new tool for observing fluctuations in the EMT/MET status of tumor cells, in real time, during different steps in the metastatic cascade.

## Conclusions

The Z-cad dual sensor provides a novel method to detect different EMT/MET states by reporting changes in two critical regulators of EMT with well-defined roles in this process. The ability of these sensors to report changes both via flow cytometry and dynamic changes in live cells using fluorescence microscopy in response to different stimuli or even in an unperturbed setting has several advantages over current methods that rely on immunostaining or lineage tracing. The lentiviral nature of the Z-cad dual sensor enables facile and stabile introduction using both in vitro and in vivo models and should aid in elucidating the mechanisms underlying EMT/MET processes in the pathology of cancer.

## Methods

### Generation of lentiviral constructs

To construct the miR-200 sensor, four oligonucleotide primers were generated containing five target sequences recognized by each of the five miR-200 family member seed sequences and annealed in vitro*.* The primers were designed so that the annealed fragment had overhangs that complement with the FUGW lentiviral vector digested with *Bsr*GI and *Eco*RI. The primers were also designed to ensure that the eGFP cDNA in the FUGW vector remained intact after ligation. The sequences of the primers are as follows:

- Mir200 sponge f1
  GTACAATAAGACATCGTTACGTCCAGTGTTAAGAGAACTTAGAGAACTTCCATCTTTACGTCCAGTGTTAAGAGAACTTAGAGAACTTTCATCATTACGTCCAGTATTA
- Mir200 sponge f2
  AGAGAACTTAGAGAACTTTCCATCATTACGGCCAGTATTAAGAGAACTTAGAGAACTTACGGCATTACGTCCAGTATTAGCTAGC
- Mir200 sponge r1
  AATTGCTAGCTAATACTGGACGTAATGCCGTAAGTTCTCTAAGTTCTCTTAATACTGGCCGTAATGATGGAAAGTTCTCTAAGTTCTCTTAATACTGGACGTAATGATGAAAGT
- Mir200 sponge r2
  TCTCTAAGTTCTCTTAACACTGGACGTAAAGATGGAAGTTCTCTAAGTTCTCTTAACACTGGACGTAACGATGTCTTACTT

The miR-200 binding sites were subcloned into the TOP-d2GFP vector. The d2GFP-miR200 sequence was then amplified with a 5′ *Bam*HI and 3′ *Nhe*I added and inserted back into FUGW to replace eGFP. The TOP-d2GFP vector was a kind gift from Drs. Wen Bu and Yi Li (Baylor College of Medicine, Houston, TX). d2GFP was subcloned into FUGW to replace eGFP and serve as a d2GFP control vector. Briefly, primers to amplify inserts were generated to contain 5′ *Bam*HI and 3′ *Nhe*I restriction sites and inserted into FUGW and FUGW-miR-200.

The *ZEB1* 3′ UTR (pRL-ZEB1) was a kind gift from Dr. Joel Neilson (Baylor College of Medicine, Houston), with permission of Dr. Gregory Goodall (University of Adelaide, Australia). The *ZEB1* 3′ UTR was amplified using primers to add *Nhe*I and *Eco*RI sites. The d2FUGW-miR-200 vector was cut with *Nhe*I and *Eco*RI and the *ZEB1* 3′ UTR was inserted.

The human E-cadherin promoter (about 1370 bp) replaced the CMV promoter into a modified pHAGE-CMV-dsRed-IRES-ZsGreen vector in which IRES-ZsGreen was previously removed.

To generate pINDUCER13-pre-miR-200c/141 (p13-miR-200c/141), miR-200c/141 from p11-miR-200c-/141 [11] and p13 were both digested using *Not*I and *Mlu*I. The miR-200c/141 sequence was inserted directly into p13, which was a kind gift from Dr. Thomas Westbrook (Baylor College of Medicine, Houston, TX).

All inserts were sequenced and verified after they were cloned into destination vectors.

### Cell culture and lentivirus transduction

The MDA-MB-231 cells were acquired from the Tissue and Cell Culture Core Laboratory at Baylor College of Medicine in Houston, TX, which originally obtained them from the American Type Culture Collection (ATCC), Manassas, VA. They were maintained in DMEM (Gibco) supplemented with 10 % fetal bovine serum (FBS) and antibiotic/antimycotic.

The T11 cells were derived from a p53 null, claudin-low mouse mammary tumor [21]. They were cultured in DMEM (Gibco) supplemented with 10 % FBS and antibiotic/antimycotic.

The BLSL12 cells were a kind gift from Dr. Matthew Ellis (Baylor College of Medicine, Houston, TX). These cells were derived from the WHIM12 claudin-low human patient derived xenograft [22]. They were cultured in RPMI-1640 supplemented with high glucose, L-glutamine, HEPES (ATCC 30-2001), 10 % FBS, and antibiotic/antimycotic.

The MCF10A cells were obtained from Dr. Dean Edwards through the Advanced Technology Core at Baylor College of Medicine in Houston, TX, which originally obtained them from ATCC. They were maintained in DMEM/F12 (Gibco) supplemented with 5 % horse serum, 20 ng/mL epidermal growth factor (EGF), 0.5 μg/mL hydrocortisone, 5 μg/mL insulin, 100 ng/mL cholera toxin, and antibiotic/antimycotic.

The HMLER cells were obtained from Dr. Sendurai Mani (MD Anderson Cancer Center, Houston, TX). They were kept under puromycin selection to maintain H-Ras (G12V) expression and were maintained in 50 % MEGM medium (Lonza CC-3051) and 50 % DMEM/F12 (Gibco) supplemented with 5 μg/mL insulin, 10 ng/mL EGF, 500 ng/mL hydrocortisone, and antibiotic/antimycotic. For EMT induction HMLER cells were treated with 5 ng/mL recombinant human TGFβ1 (R&D Systems, 240-B). TGFβ1 was prepared according to the manufacturer’s protocol.

All cell lines were transduced with lentiviral fluorescent sensors at a MOI = 5. HMLER and MCF10A cells were sorted for double positive cells to ensure provirus presence in all cells. MDA-MB-231, T11, and BLSL12 cells were transduced with p13-miR-200/141 (see below) and selected for 3 days in 2 μg/mL puromycin. To induce miR-200c/141, cells were treated with 2 μg/mL doxycycline for the indicated time periods.

Decitabine (5-aza-2’-deoxycytidine) was purchased from Sigma Aldrich (#A3656) and reconstituted in DMSO. The medium was changed daily during treatment studies.

### Tumorsphere assays

The HMLER tumorsphere medium was made using MEGM medium (Lonza CC-3051) without BPE with the following supplements: 20 ng/mL bFGF, 10 ng/mL EGF, 4 ug/mL heparin, and 1 % methyl cellulose (to prevent aggregation).

The cells were sorted according to the fluorescent sensor or CD24/CD44 expression as indicated in the text. Once sorted, the cells were centrifuged, washed two times in mammosphere medium without BPE, and counted. 1000 cells per well were plated in 100 μL mammosphere medium (5 wells per group, per replicate) in a low-attachment 96-well plate. Tumorsphere medium (100 μL) was added every 3 days. Twelve days after seeding, tumorspheres (>75 μm) were quantified. Five technical replicate experiments were performed for each of the three biological replicates per group.

### Western blot

Tumor cells were harvested by mechanical scraping and resuspended in RIPA buffer containing protease and phosphatase inhibitors (Roche), placed on ice for 30 min, and resuspended periodically. The lysed cells were centrifuged and supernatants were isolated. The protein concentrations were determined using a Bradford assay, loaded and run on a 10 % Tris-glycine SDS-polyacrylamide gel. The transfers were performed overnight at 4 °C. The following antibodies and conditions were used: mouse monoclonal Anti-β-Actin (Sigma-Aldrich, Clone AC-15, A5441) 1:5000 in 5 % milk in TBS-T, rabbit monoclonal Anti-E-cadherin (Cell Signaling Technology, Clone 24E10, 3195S) 1:1000 in 5 % BSA in TBS-T, rabbit polyclonal Anti-ZEB1 (Santa Cruz Biotechnology, SC-25388) 1:500 in 5 % milk in TBS-T.

### RNA isolation and qRT-PCR

RNA isolation was performed using the miRNeasy Mini Kit (Qiagen) and treated with the RNase-Free DNase (Qiagen).

Reverse transcription of mRNAs was performed using a High-Capacity RNA-to-cDNA Kit (Thermo Fisher Scientific). 100 ng total RNA was used for each reaction. cDNA was diluted to 1 ng/μL prior to qPCR. 2 ng of cDNA was added to each well (each sample was performed in triplicate as technical replicates). The following TaqMan probes and primers from Thermo Fisher were used for qRT-PCR: 18 s (Hs99999901_s1), human Zeb1 (Hs00232783_m1), mouse Zeb1 (Mm00495564_m1), human CDH1 (Hs01013959_m1, mouse Cdh1 (Mm01247357_m1), and human vimentin (Hs00185584_m1).

Reverse transcription (RT) PCR for miRNAs was performed using the TaqMan Small RNA Assays (Thermo Fisher) with corresponding miRNA assays as listed below. 10 ng of total RNA was added to each reaction and RT was performed according to the manufacturer’s protocol. The following TaqMan RT primers and qPCR probes and primers from Thermo Fisher were used for qRT-PCR: U6 snRNA (001973), miR-200b (002251), and miR-200c (002300).

TaqMan Gene Expression Master Mix (Thermo Fisher) was used for all qPCR reactions. qPCR was performed on a Step One Plus Real-Time PCR machine. For non-detectable expression during qPCR, Ct values were set to 39.

### Microscopy

All fluorescent images were taken using a Nikon A1-R confocal microscope with NIS Elements acquisition software and analyzed using FIJI Software. IBIDI μ-Dish 35 mm low (#80136) was used for imaging T11 and BLSL12 cells and IBIDI μ-Slide 8 well Grid-500 (#80826-500G) was used for MDA-MB-231 time-lapse microscopy.

Brightfield images were acquired using a Zeiss Observer.A1 inverted microscope using AxioVision Microscopy Software.

### Cytospin and co-immunofluorescence

Cells isolated by FACS were collected and about 30,000 cells were cytospun onto glass slides. Cells were fixed with 4 % paraformaldehyde for 20 min at room temperature followed by three washes in phosphate-buffered saline (PBS) for 5 min each. Slides were stored in 70 % ethanol at 4 °C overnight (or until use, no more than 1 week).

For immunostaining, cells were rinsed twice with PBS followed by one wash in PBS with Tween-20 (0.5 % Triton X-100 to ensure quenching of endogenous fluorescence), rinsed twice in PBS, and blocked for 1 hour using 5 % goat serum + M.O.M. block (Mouse on Mouse M.O.M. basic kit, Vector Labs, BMK-2202). Samples were washed once in PBS followed by incubation for 5 min with 5 % goat serum + M.O.M. diluent. Primary antibodies (1:200 dilution for each in 5 % goat serum + M.O.M. diluent) were added to cells for 1 hour at room temperature. Rabbit monoclonal antibody against vimentin (Cell Signaling 5741) and mouse monoclonal antibody against pan-cytokeratin (Sigma C2562) were used followed by three washes with PBS (no primary antibody was added to the diluent for secondary only control). Secondary antibodies were added in PBS for 1 hour at room temperature (1:1000 dilution for both secondary antibodies (Goat anti-Rabbit AlexaFluor 488 – Thermo Fisher A11034, Goat anti-Mouse AlexaFluor 594 – Thermo Fisher A11005) and washed four times with PBS. A final wash containing 1 drop/mL DAPI in PBS (NucBlue Fixed Cell Ready Probes, Thermo Fisher R37606) was performed. Slides were mounted using Aqua-Poly/Mount (Polysciences, Inc. 18606-20).

### Flow cytometry

Briefly, all cells were harvested for flow cytometry using the following protocol. Cells were trypsinized and neutralized with growth medium containing 10 % FBS and the medium was removed. Cells were resuspended in HBSS+ (HBSS + 2 % FBS + HEPES buffer) and passed through a 40-μm mesh filter.

For antibody staining of HMLER cells, anti-human CD24-APC (eBioscience, clone eBioSN3, ref. 17-0247-42) and anti-human/mouse CD44-eFluor 450 (eBioscience, clone IM7, 48-0441-82) were diluted in HBSS+ at 1:50 dilution and used to resuspend cells. Cells were incubated for 30 min at 4 °C, rinsed twice in HBSS+, resuspended in HBSS+, and filtered through a 40-μm mesh filter.

Flow cytometry analysis was performed on BD LSR Fortessa. Sorting was performed on BD AriaII. FlowJo was used for the analysis.

### Statistical analysis

All bar graphs indicate the mean value. All error bars shown represent the standard deviation. Paired or unpaired Student’s *t* tests were performed as indicated for all statistical analyses.

## Abbreviations

CK, pan-cytokeratin; CMV, cytomegalovirus; CSC, cancer stem cell; DOX, doxycycline; EMT, epithelial to mesenchymal transition; FACS, fluorescent activated cell sorting; GFP, green fluorescent protein; MET, mesenchymal to epithelial transition; miR, microRNA; NCSC, non-cancer stem cell; PDX, patient-derived xenograft; RFP, red fluorescent protein; UbC, ubiquitin C UTR, untranslated region.

## Additional files

Additional file 1: Figure S1. miR-200 sensor construct and validation; Z-cad sensor validation. A) Five miR-200 family binding sites were placed downstream of d2GFP in the FUGW lentiviral expression vector. B) Fluorescent microscopy of T11 cells containing the Z-cad dual sensor after 4 days of doxycycline treatment. C) Flow cytometry analysis of d2GFP-200 or control d2GFP expression and Ecad-RFP upon miR-200c/141 induction after 4 days of 2 μg/mL doxycycline treatment in the indicated cell lines (*n* = 3 biological replicates per group). D) Flow cytometry analysis of BLSL12 breast cancer cells containing the indicated sensors. miR-200c/141 was induced with 2 μg/mL doxycycline for 4 days. E) Fluorescent confocal microscopy of BLSL12 cells containing Z-cad dual sensor after 4 days of doxycycline treatment. Scale bar = 20 μm. (TIF 4770 kb) Additional file 2: Figure S2. Z-cad sensor loses GFP expression early and gains RFP expression later upon miR-200c induction. All collected time points for time-lapse microscopy (from Fig. 1c) of identical grids within cell culture plate are shown for each treatment group. A) –DOX control. B) 2 μg/mL DOX treatment to induce miR-200c. All time points after DOX treatment are indicated. Scale bars = 50 μm. (TIF 9490 kb) Additional file 3: Figure S3. Decitabine treatment elicits RFP gain and GFP loss using the Z-cad sensor. A) Flow cytometry analysis indicating RFP^+^ cells (*above horizontal line*) and GFP low cells (*left of vertical line*). GFP low/RFP^+^ are cells falling into *top left quadrant*. Quantitation is shown in B). Unpaired Student’s *t* test was performed (*n* = 3 biological replicates for vehicle and 500 nM; *n* = 2 for 100 nM). * *p* value < 0.05, ** *p* value < 0.01. (TIF 1358 kb) Additional file 4: Figure S4. The Z-cad sensor enables isolation of EMT-like MCF10A cells. A) FACS was performed on parental MCF10A cells containing the Z-cad dual sensor to isolate GFP^hi^RFP^low/neg^ cells, which comprise the EMT signature. Bulk^–EMT^ group comprises all other cells. B) RNA from each group collected in A) was isolated and qRT-PCR performed for the indicated genes. Bulk^–EMT^ cell values were set to 1.0 for each gene and analyzed using a paired Student’s *t* test (*n* = 3 biological replicates). (TIF 1316 kb) Additional file 5: Figure S5. The Z-cad sensor can separate an EMT-like cellular subpopulation during TGFβ1 treatment. A) RNA analysis of HMLER cells isolated using EMT or Bulk^–EMT^ fluorescence signatures during TGFβ1 treatment by qRT-PCR at indicated time points (*n* = 2 biological replicates for 6 and 12 days; *n* = 3 biological replicates for 18 and 24 days). Bulk^–EMT^ cell values were set to 1.0 for each gene. Paired Student’s *t* test was performed. * *p* value < 0.05, ** *p* value < 0.01. B) Cytospun HMLER cells collected from EMT and Bulk^–EMT^ fluorescence groups at 24 days of TGFβ1 treatment. Immunofluorescence for pan-CK and vimentin was performed. (TIF 2067 kb) Additional file 6: Figure S6. Z-cad population gates and their CD24/CD44 profiles. A) Flow cytometry analysis was used to gate HMLER cells based on the indicated Z-cad expression profiles (in *red boxes*). Gated cells from each population were subsequently analyzed for CD24 and CD44 expression as shown in B). (TIF 1218 kb) Additional file 7: Supporting Data. (XLSX 51 kb)
