# Supplementary figures and images for: The Z-cad dual fluorescent sensor detects dynamic changes between the epithelial and mesenchymal cellular states
Source: BMC Biol. 2016 Jun 17;14:47. doi: 10.1186/s12915-016-0269-y (PMC4912796; doi:10.1186/s12915-016-0269-y)

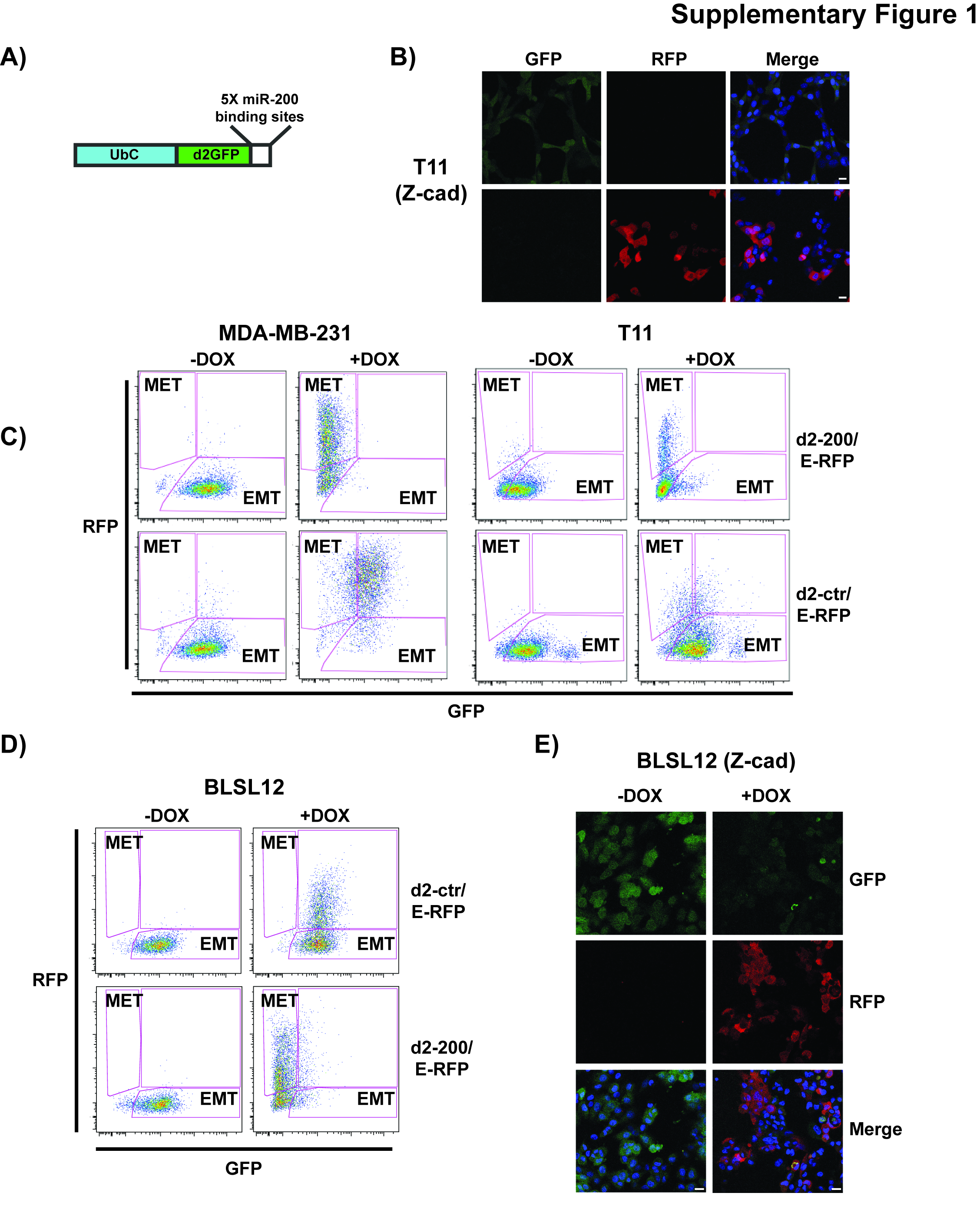

Supplement: Additional file 1: Figure S1. — miR-200 sensor construct and validation; Z-cad sensor validation. A) Five miR-200 family binding sites were placed downstream of d2GFP in the FUGW lentiviral expression vector. B) Fluorescent microscopy of T11 cells containing the Z-cad dual sensor after 4 days of doxycycline treatment. C) Flow cytometry analysis of d2GFP-200 or control d2GFP expression and Ecad-RFP upon miR-200c/141 induction after 4 days of 2 μg/mL doxycycline treatment in the indicated cell lines (n = 3 biological replicates per group). D) Flow cytometry analysis of BLSL12 breast cancer cells containing the indicated sensors. miR-200c/141 was induced with 2 μg/mL doxycycline for 4 days. E) Fluorescent confocal microscopy of BLSL12 cells containing Z-cad dual sensor after 4 days of doxycycline treatment. Scale bar = 20 μm. (TIF 4770 kb) [file 12915_2016_269_MOESM1_ESM.tif]

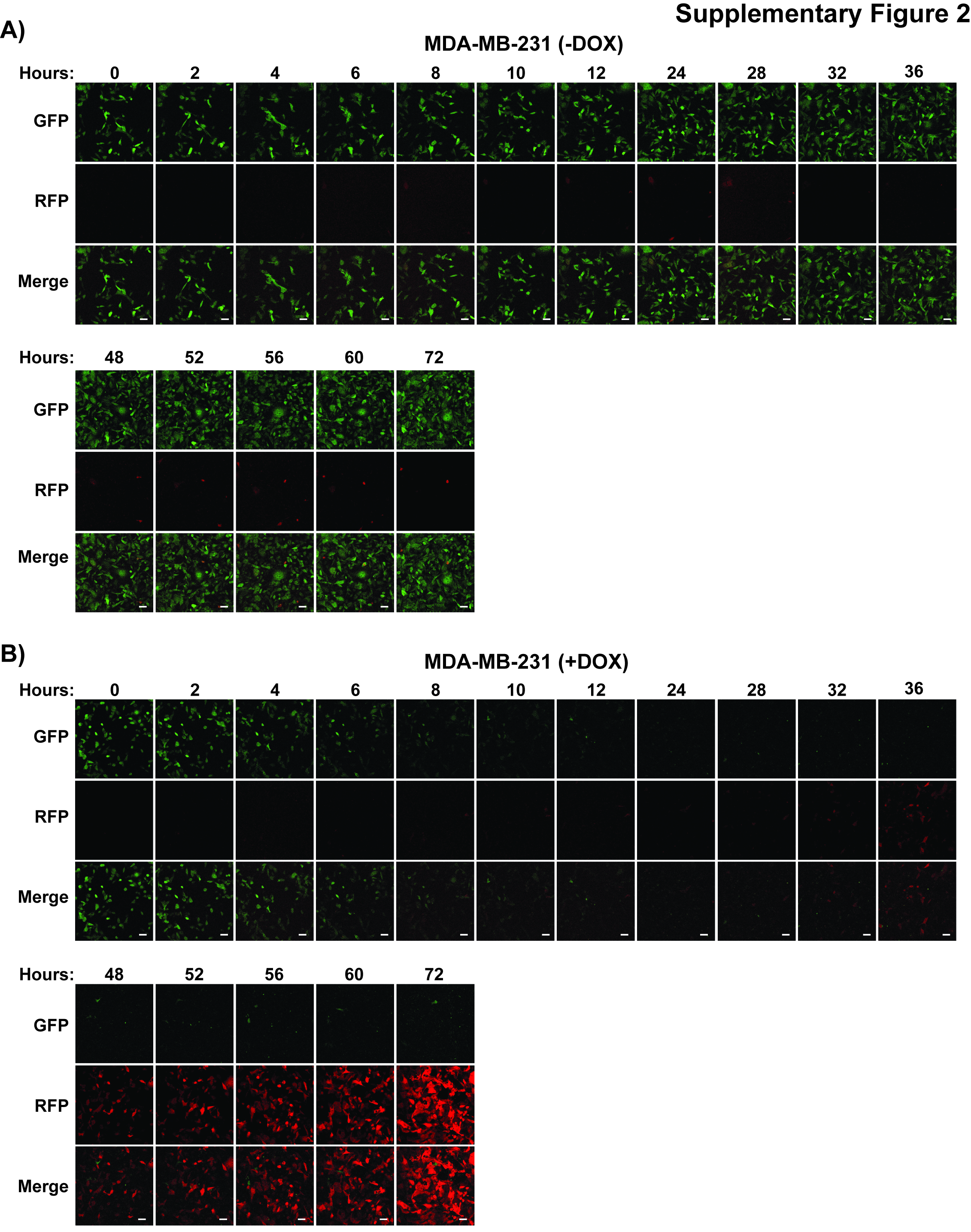

Supplement: Additional file 2: Figure S2. — Z-cad sensor loses GFP expression early and gains RFP expression later upon miR-200c induction. All collected time points for time-lapse microscopy (from Fig. 1c) of identical grids within cell culture plate are shown for each treatment group. A) –DOX control. B) 2 μg/mL DOX treatment to induce miR-200c. All time points after DOX treatment are indicated. Scale bars = 50 μm. (TIF 9490 kb) [file 12915_2016_269_MOESM2_ESM.tif]

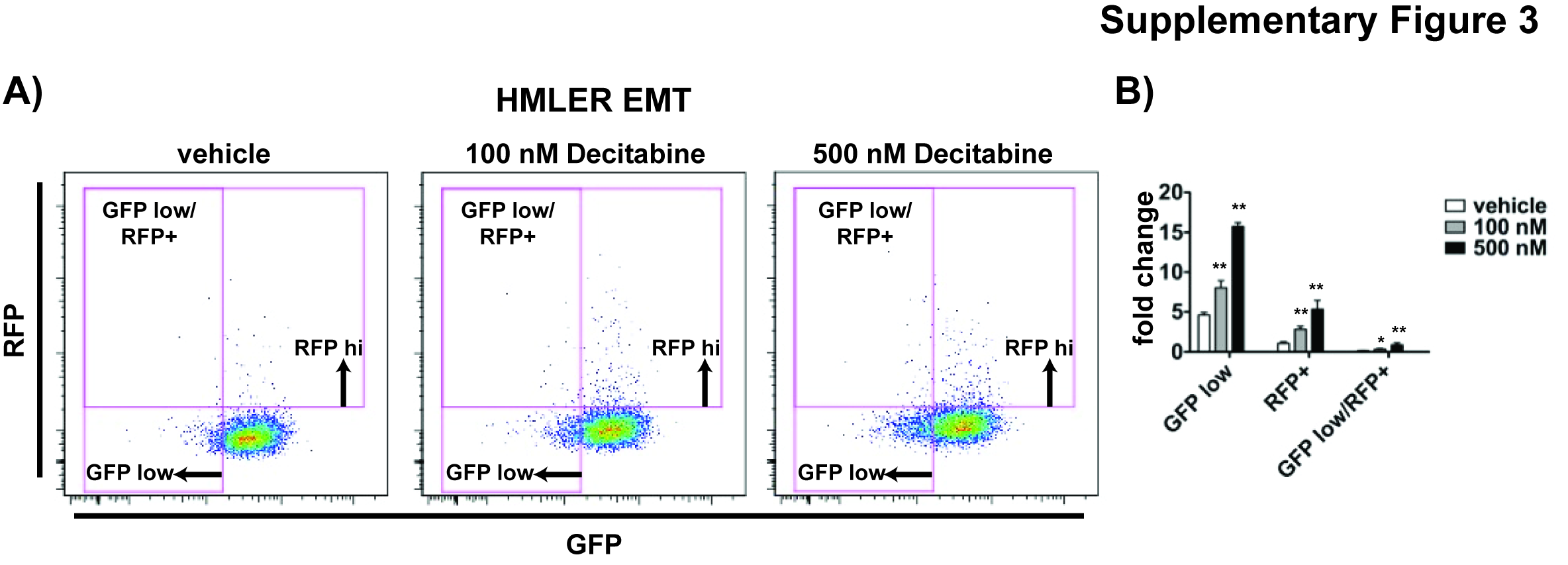

Supplement: Additional file 3: Figure S3. — Decitabine treatment elicits RFP gain and GFP loss using the Z-cad sensor. A) Flow cytometry analysis indicating RFP+ cells (above horizontal line) and GFP low cells (left of vertical line). GFP low/RFP+ are cells falling into top left quadrant. Quantitation is shown in B). Unpaired Student’s t test was performed (n = 3 biological replicates for vehicle and 500 nM; n = 2 for 100 nM). * p value < 0.05, ** p value < 0.01. (TIF 1358 kb) [file 12915_2016_269_MOESM3_ESM.tif]

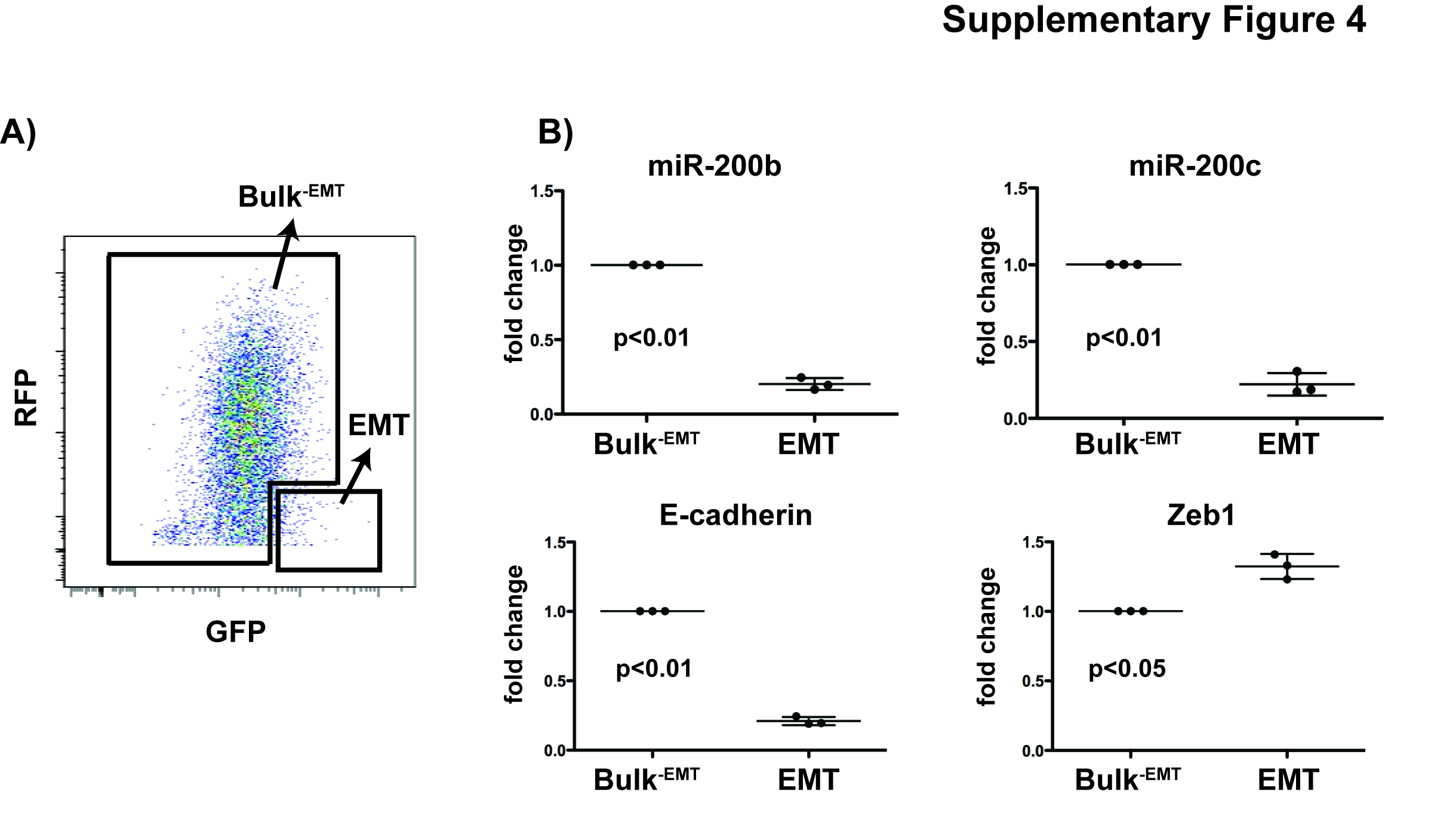

Supplement: Additional file 4: Figure S4. — The Z-cad sensor enables isolation of EMT-like MCF10A cells. A) FACS was performed on parental MCF10A cells containing the Z-cad dual sensor to isolate GFPhiRFPlow/neg cells, which comprise the EMT signature. Bulk–EMT group comprises all other cells. B) RNA from each group collected in A) was isolated and qRT-PCR performed for the indicated genes. Bulk–EMT cell values were set to 1.0 for each gene and analyzed using a paired Student’s t test (n = 3 biological replicates). (TIF 1316 kb) [file 12915_2016_269_MOESM4_ESM.tif]

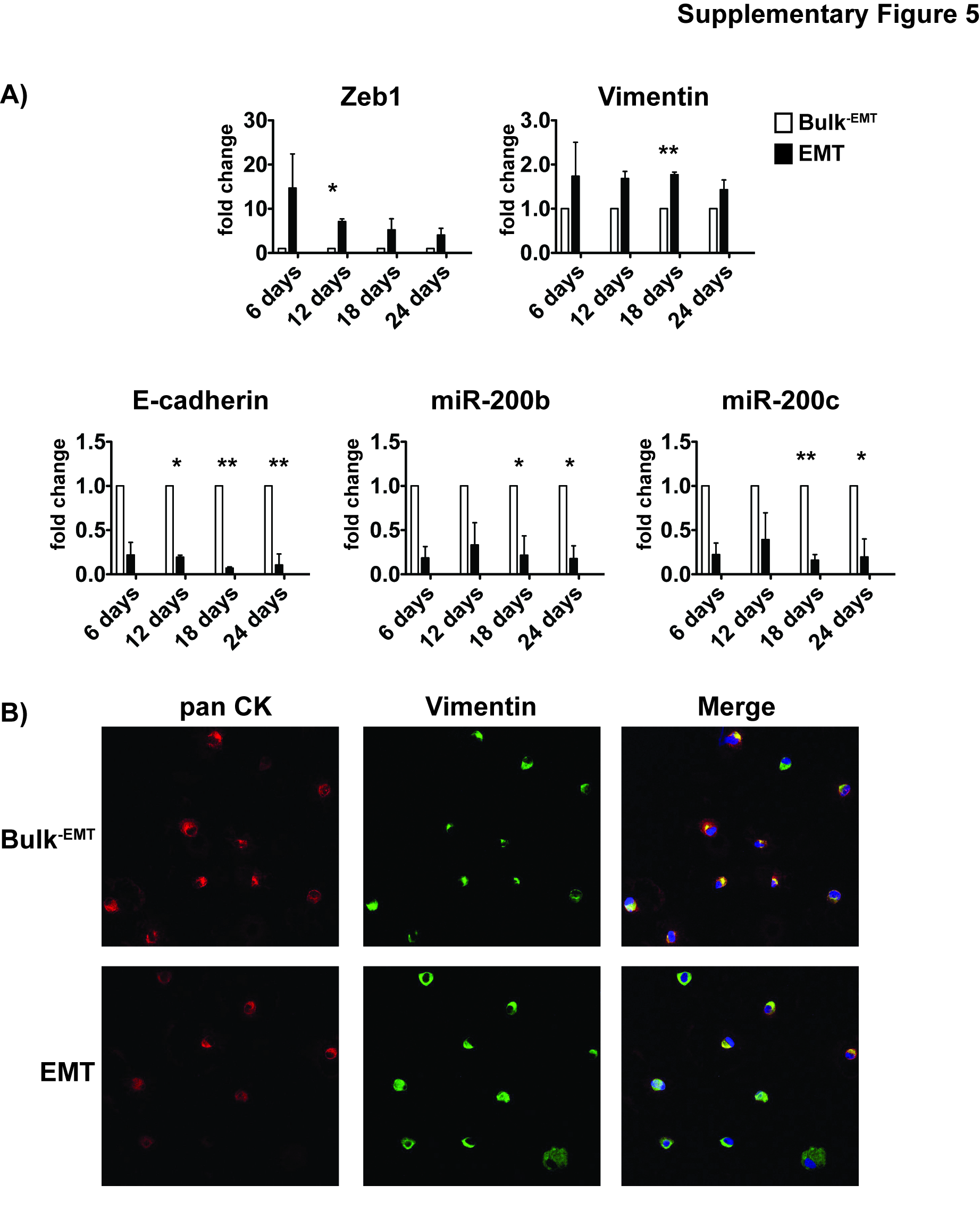

Supplement: Additional file 5: Figure S5. — The Z-cad sensor can separate an EMT-like cellular subpopulation during TGFβ1 treatment. A) RNA analysis of HMLER cells isolated using EMT or Bulk–EMT fluorescence signatures during TGFβ1 treatment by qRT-PCR at indicated time points (n = 2 biological replicates for 6 and 12 days; n = 3 biological replicates for 18 and 24 days). Bulk–EMT cell values were set to 1.0 for each gene. Paired Student’s t test was performed. * p value < 0.05, ** p value < 0.01. B) Cytospun HMLER cells collected from EMT and Bulk–EMT fluorescence groups at 24 days of TGFβ1 treatment. Immunofluorescence for pan-CK and vimentin was performed. (TIF 2067 kb) [file 12915_2016_269_MOESM5_ESM.tif]

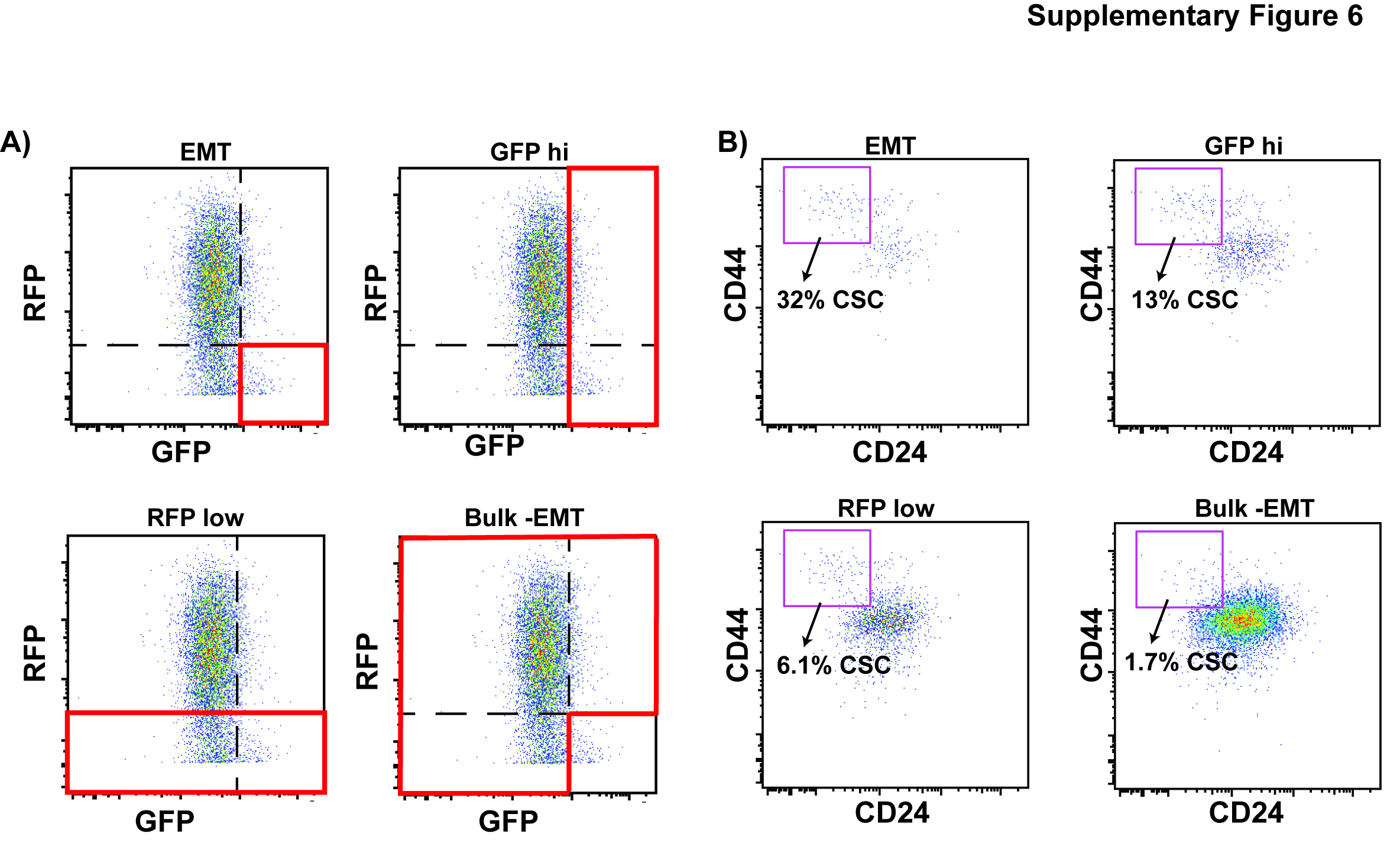

Supplement: Additional file 6: Figure S6. — Z-cad population gates and their CD24/CD44 profiles. A) Flow cytometry analysis was used to gate HMLER cells based on the indicated Z-cad expression profiles (in red boxes). Gated cells from each population were subsequently analyzed for CD24 and CD44 expression as shown in B). (TIF 1218 kb) [file 12915_2016_269_MOESM6_ESM.tif]
